# Biomechanical Mapping of the Female Pelvic Floor: Prolapse versus Normal Conditions

**DOI:** 10.4236/ojog.2018.810093

**Published:** 2018-08-31

**Authors:** Vladimir Egorov, S. Abbas Shobeiri, Peter Takacs, Lennox Hoyte, Vincent Lucente, Heather van Raalte

**Affiliations:** 1 Artann Laboratories, Trenton, USA; 2 INOVA Fairfax Hospital, Falls Church, USA; 3 Eastern Virginia Medical School, Norfolk, USA; 4 The Pelvic Floor Institute, Tampa, USA; 5 The Institute for Female Pelvic Medicine & Reconstructive Surgery, Allentown, USA; 6 Princeton Urogynecology, Princeton, USA

**Keywords:** Biomechanical Mapping, Female Pelvic Floor, Prolapse, Tissue Elasticity, Pelvic Support, Pelvic Function, Tactile Imaging, Elastography

## Abstract

**Background:**

Quantitative biomechanical characterization of pelvic supportive structures and functions *in vivo* is thought to provide insight into pathophysiology of pelvic organ prolapse (POP). An innovative approach—vaginal tactile imaging—allows biomechanical mapping of the female pelvic floor to quantify tissue elasticity, pelvic support, and pelvic muscle functions. The Vaginal Tactile Imager (VTI) records high definition pressure patterns from vaginal walls under an applied tissue deformation and during pelvic floor muscle contractions.

**Objective:**

To explore an extended set of 52 biomechanical parameters for differentiation and characterization of POP relative to normal pelvic floor conditions.

**Methods:**

96 subjects with normal and POP conditions were included in the data analysis from multi-site observational, case-controlled studies; 42 subjects had normal pelvic floor conditions and 54 subjects had POP. The VTI, model 2S, was used with an analytical software package to calculate automatically 52 biomechanical parameters for 8 VTI test procedures (probe insertion, elevation, rotation, Valsalva maneuver, voluntary muscle contractions in 2 planes, relaxation, and reflex contraction). The groups were equalized for subject age and parity.

**Results:**

The ranges, mean values, and standard deviations for all 52 VTI parameters were established. 33 of 52 parameters were identified as statistically sensitive (*p* < 0.05; *t*-test) to the POP development. Among these 33 parameters, 11 parameters show changes (decrease) in tissue elasticity, 8 parameters show deteriorations in pelvic support and 14 parameters show weakness in muscle functions for POP *versus* normal conditions.

**Conclusions:**

The biomechanical mapping of the female pelvic floor with the VTI provides a unique set of parameters characterizing POP *versus* normal conditions. These objectively measurable biomechanical transformations of pelvic tissues, support structures, and functions under POP may be used in future research and practical applications.

## 1. Introduction

Recent survey identified the highest priority research questions pertaining to pathophysiology and treatments of pelvic organ prolapse (POP); according to it, mechanistic research on pelvic supportive structures, clinical trials to optimize outcomes after POP surgery and evidence-based quality measures for POP outcomes are among the major focus areas [1]. In vaginal prolapse surgery, about 20% of procedures are performed for recurrent POP. There are not many other fields with such poor surgical outcomes [2].

Many pelvic floor disorders, including POP, stress urinary incontinence (SUI), sexual dysfunction, congenital anomalies, and others, are clearly manifested in the mechanical properties of pelvic organs. Therefore, biomechanical mapping of a response to applied pressure or load within the pelvic floor opens new possibilities in biomechanical assessment and monitoring of pelvic floor conditions. The newly developed vaginal tactile imaging allows biomechanical mapping of the female pelvic floor including assessment of tissue elasticity, pelvic support, and pelvic muscle functions in high definition [3] [4] [5] [6].

Previously, we reported the intra- and inter-observer reproducibility of vaginal tactile imaging [7] and proposed interpretation of biomechanical mapping of the female pelvic floor [8]. The new mechanistic parameters were introduced for assessment of the vaginal [9] and pelvic floor conditions [10].

The objective of this study is to identify an extended set of Vaginal Tactile Imager (VTI) parameters which would comprehensively characterize the pelvic floor tissues, support structures and functions contributing to the POP development, and to establish their ranges for visualization of every biomechanical parameter acquired for specific patient conditions.

## 2. Materials and Methods

### 2.1. Definitions

*Tactile Imaging* is a medical imaging modality translating the sense of touch into a digital image [10]. The tactile image is a function of *P* (*x, y, z*), where *P* is the pressure on soft tissue surface under applied deformation and *x, y* and *z* are the coordinates where *P* was measured. The tactile image is a pressure map on which the direction of tissue deformation must be specified.

*Functional Tactile Imaging* translates muscle activity into dynamic pressure pattern *P* (*x, y, t*) for an area of interest, where *t* is time and *x* and *y* are coordinates where pressure *P* was measured. It may include: 1) muscle voluntary contraction, 2) involuntary reflex contraction, 3) involuntary relaxation, and 4) specific maneuvers.

BiomechanicalMapping=TactileImaging+FunctionalTactileImaging

A tactile imaging probe has a pressure sensor array mounted on its face that acts similar to human fingers during a clinical examination, deforming the soft tissue and detecting the resulting changes in the pressure pattern on the surface. The sensor head is moved over the surface of the tissue to be studied, and the pressure response is evaluated at multiple locations along the tissue. The results are used to generate 2D/3D images showing pressure distribution over the area of the tissue under study.

Generally, an inverse problem solution for tactile image *P* (*x, y, z*) would allow the reconstruction of tissue elasticity distribution (*E*) as a function of the same coordinates *E* (*x, y, z*). Unfortunately, the inverse problem solution is hardly possible for most real objects because it is a non-linear and ill-posed problem. However, the tactile image *P* (*x, y, z*) per se reveals tissue or organ anatomy and elasticity distribution because it maintains the stress-strain relationship for deformed tissue [11] [12]. Thus the spatial gradients *∂P* (*x, y, z*)/*∂x*, *∂P* (*x, y, z*)/*∂y*, and *∂P* (*x, y, z*)/*∂z* can be used in practice for soft tissue elasticity mapping, despite structural and anatomical variations [3].

### 2.2. Vaginal Tactile Imager

**Figure 1.**
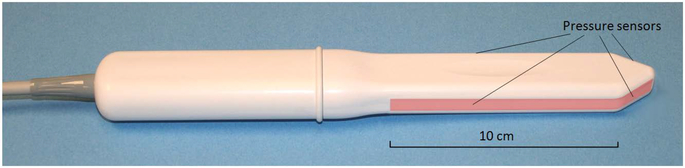
Vaginal probe. Pressure sensors are aligned on the outer surfaces of the probe (highlighted in the image).

The VTI, model 2S (Advanced Tactile Imaging, Inc., NJ), was used in all test procedures. The VTI probe, as shown in Figure 1, is equipped with 96 pressure (tactile) sensors spaced at 2.5 mm consecutively on both sides of the probe, an orientation sensor, and temperature controllers to provide the probe temperature close to a human body before the examination. During the clinical procedure, the probe is used to acquire pressure responses from two opposite vaginal walls along the vagina. The VTI data are sampled from the probe sensors and displayed on the VTI monitor in real time. The resulting pressure maps (tactile images) of the vagina integrate all the acquired pressure and positioning data for each of the pressure sensing elements. Additionally, the VTI records the dynamic contraction for pelvic floor muscles with resolution of 1 mm. A lubricating jelly is used for patient comfort and to provide reproducible boundary/contact conditions with deformed tissues.

This VTI probe allows 3 – 15 mm tissue deformation at the probe insertion (Tests 1), 20 – 45 mm tissue deformation at the probe elevation (Test 2), 5 – 7mm deformation at the probe rotation (Test 3) and recording of dynamic responses at pelvic muscle contractions (Tests 4 – 8). The probe maneuvers in Tests 1 – 3 allow accumulation of multiple pressure patterns from the tissue surface to compose an integrated tactile image for the investigated area using a proprietary image composition algorithm similar to the imaging of the prostate and breast [11] [12]. The spatial gradients *∂P* (*x, y*)/*∂y* for anterior and posterior compartments are calculated within the acquired tactile images in test 1 and 2; y-coordinate is directed orthogonally from the vaginal channel, x-coordinate is located on the vaginal channel. The VTI software includes data analysis tools and reporting functions. It visualizes the anatomy, pressure maps, and calculates (automatically) 52 VTI parameters for eight test procedures. The VTI examination procedure consists of eight tests: 1) probe insertion, 2) elevation, 3) rotation, and 4) Valsalva maneuver, 5) voluntary muscle contraction, 6) voluntary muscle contraction (left *versus* right side), 7) involuntary relaxation, and 8) reflex muscle contraction (cough). Tests 1 – 5 and 7 – 8 provide data for anterior/posterior compartments; test 6 provides data for left/right sides (see Table 1).

The VTI absolute measurement accuracy is as follows: ±0.2 kPa within 10 kPa range, ±0.5 kPa at 25 kPa, ±1.0 kPa at 60 kPa. The VTI relative pressure measurement accuracy lies in the range between ±0.05 kPa to ±0.1 kPa. The VTI pressure measurement resolution is 0.001 kPa. The VTI absolute measurement accuracy for probe orientation is ±0.5 degree and ±0.1˚C for measuring the temperature inside the probe on the surface of the pressure sensors. The VTI probe was calibrated immediately before every subject examination; it was cleaned and disinfected between the patients.

### 2.3. Biomechanical Mapping Parameters

Table 2 lists 52 biomechanical parameters being calculated for every 96 participating subject based on VTI data recorded in tests 1 – 8. Anatomical assignment of the targeting/contributing pelvic structures into the specified parameters is based on already published data [8] [13] [14] [15] [16] [17].

**Figure 2.**
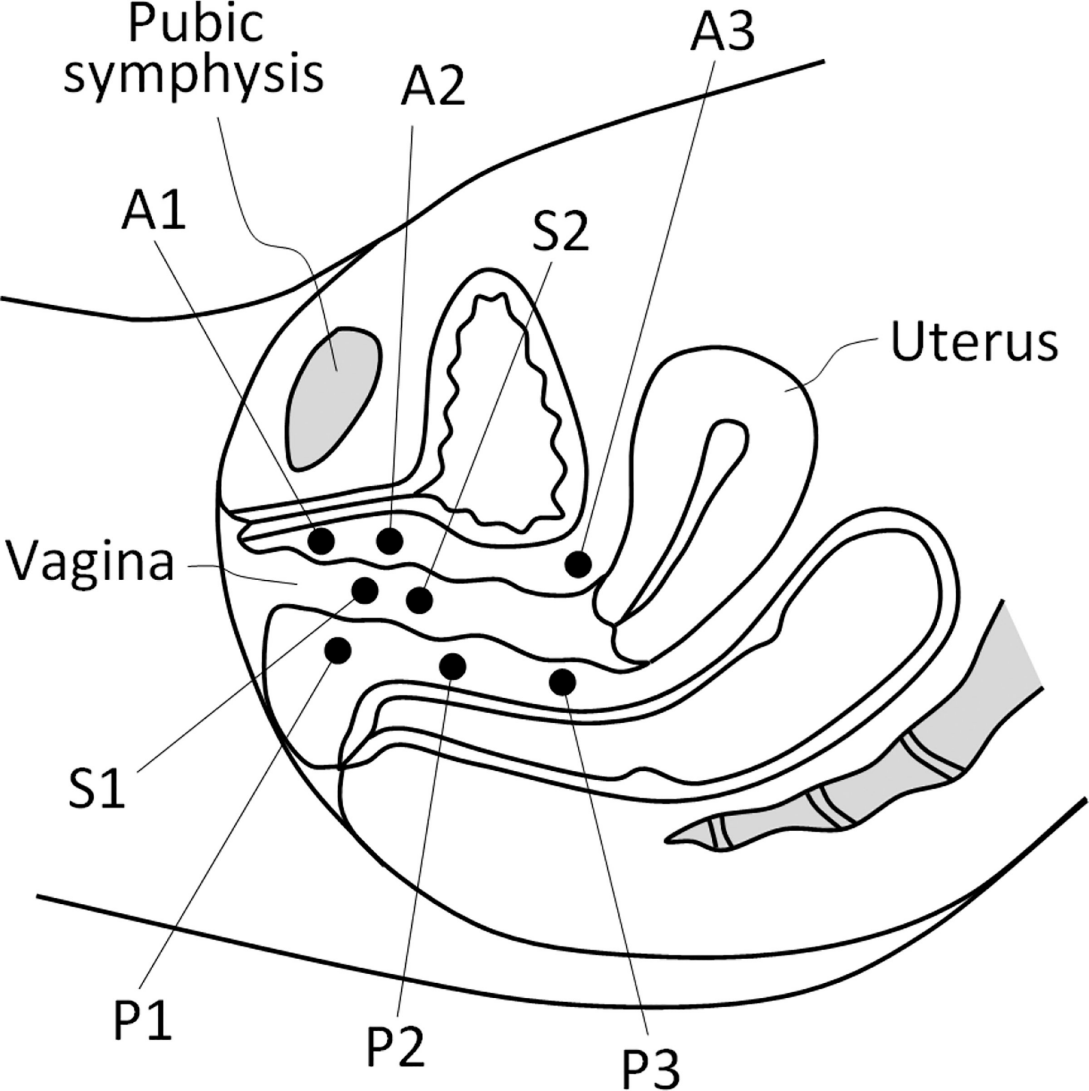
Locations of the VTI parameters within the pelvic floor. A1-A3 are in anterior compartment (Test 2), P1-P3 in posterior compartment (Test 2), and S1, S2 are in lateral compartments (left and right sides, Test 3).

Figure 2 shows the locations of the measured VTI parameters for test 2 and 3 in mid-sagittal plane of the female pelvic floor. Location A1 represents pubic bone, A2 urethra, A3 anterior part connected with cervix, P1 perineal body (Level III support), P2 mid posterior part (Level II support), P3 upper posterior part (Level I support), S1 distal part, and S2 mid-vaginal part.

### 2.4. Population Description

96 subjects with normal and POP conditions were included in the data analysis from multi-site observational, case-controlled studies with 243 enrolled subjects to date (clinical trials identifiers NCT02294383 and NCT02925585). Inclusion criteria: subject is female of 21 years or older, no prior pelvic floor surgery, and normal pelvic floor conditions or POP (any stage). Additional inclusion criteria for the analyzed data set were: all 8 VTI tests were completed, and case report and VTI data were verified. Exclusion criteria: active skin infection or ulceration within the vagina; presence of a vaginal septum; active cancer of the colon, rectum wall, cervix, vaginal, uterus or bladder; ongoing radiation therapy for pelvic cancer; impacted stool; significant pre-existing pelvic pain including levator ani syndrome, severe vaginismus or vulvadynia; severe hemorrhoids; significant circulatory or cardiac conditions that could cause excessive risk from the examination as determined by attending physician; and current pregnancy. The subject age, height, weight, and parity distribution data are present in Table 3. Prior to the VTI examination, a standard physical examination was performed, including a bimanual pelvic examination and Pelvic Organ Prolapse Quantification (POP-Q) [18]. The pelvic floor conditions were categorized by prolapse staging based on maximum stage from anterior, posterior, and uterine prolapse. Employing this approach, we found that 42 subjects had normal pelvic floor conditions (no POP, no SUI) and 54 had POP conditions (two with pelvic organ prolapse Stage I, 23 with Stage II, and 29 with Stage III). Among subjects with POP conditions we found 29 suffered from SUI, 10 had urinary urgency, and three had fecal incontinence. None of the analyzed subjects had a prior history of pelvic floor surgery. The basic demograpphic data (age, parity, weight) for both normal and POP groups are presented in Table 3. The clinical protocol was approved by the Institutional Review Board (Western IRB and local where required) and all women provided written informed consent to be enrolled into the study. This clinical research was done in compliance with the Health Insurance Portability and Accountability Act. The VTI examination data for eight ests (see Table 1) were obtained and recorded at the time of the scheduled routine urogynecologic visits.

Total study workflow comprised of the following steps: 1) Recruiting women who routinely undergo vaginal examination as a part of their diagnostic treatment of concerned areas; 2) Acquisition of clinical diagnostic information related to the studied cases by standard clinical means; 3) Performing a VTI examination in lithotomic position; 4) Analyzing VTI data and assessment of the VTI parameters for pelvic floor characterization for normal *versus* POP conditions.

### 2.5. Statistical Analysis

52 biomechanical parameters were calculated automatically per each of the 96 analyzed VTI examinations or cases (one VTI examination per each subjects). In some rare cases the parameter calculation required a manual correction of the anatomical location where the parameters must be calculated. Unpaired *t*-test (normal *versus* POP group) was completed per parameter to determine whether the parameter showed dependence on the pelvic floor conditions. For visual evaluation of the analyzed clinical data distributions we used notched boxplots [19] showing a confidence interval for the median value (central horizontal line), 25% and 75% quartiles. The spacing between the different parts of the box helps to compare variance. The boxplot also determines skewness (asymmetry) and outlier (cross). The intersection or divergence of confidence intervals for two patient samples is a visual analog of the *t-test*. The MATLAB (MathWorks, MA) statistical functions were used for the data analysis.

## 3. Results

First, the VTI visual data for all eight tests are displayed in Figures 3–10 to illustrate the approach and location used in calculating the biomechanical parameters.

**Figure 3.**
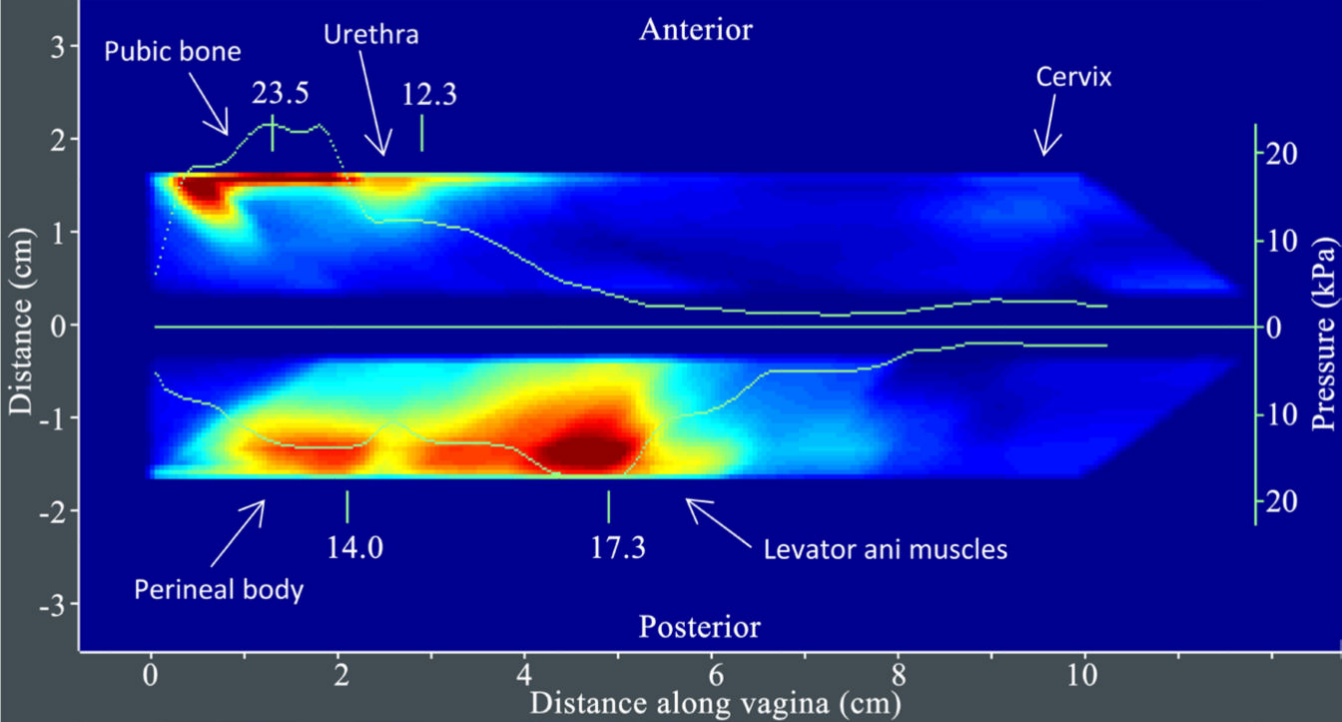
A tactile image acquired during the VTI probe insertion (Test 1) with anatomical landmarks and maximum pressure graphs (green lines, kPa) along anterior and posterior compartments.

**Figure 4.**
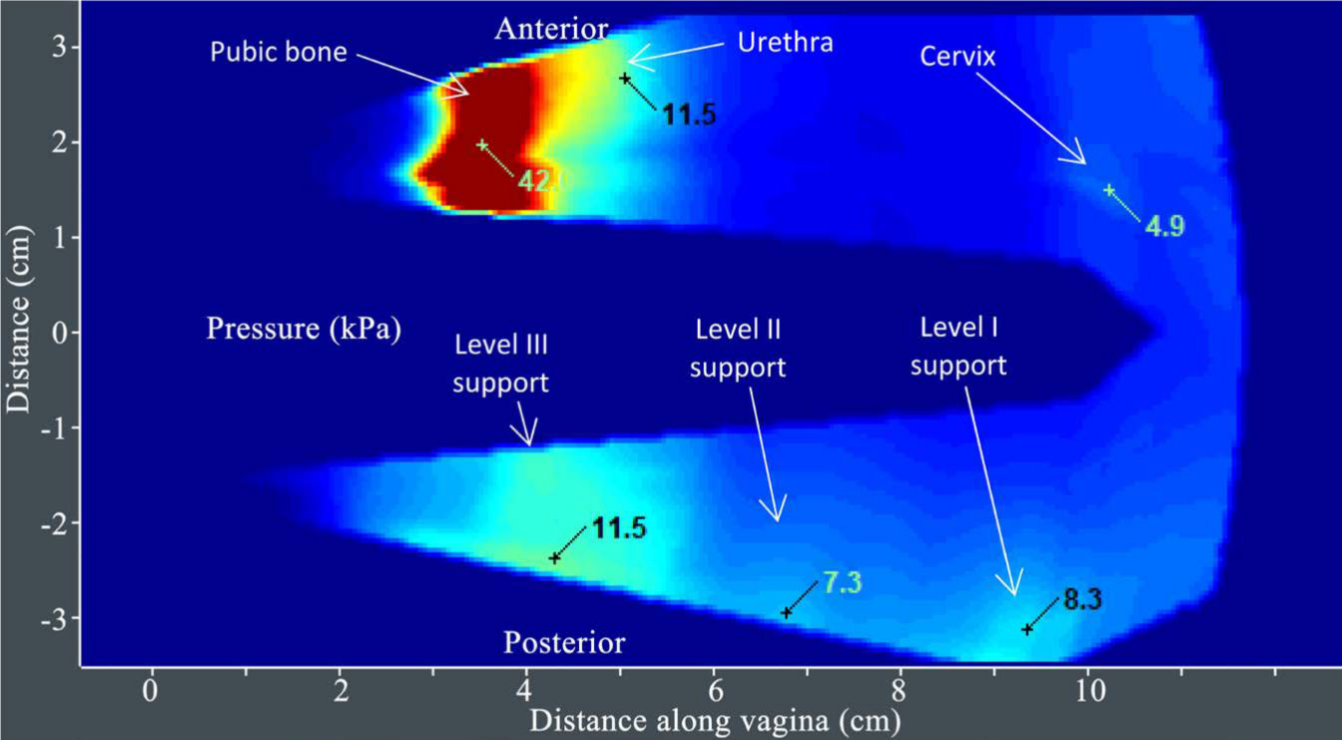
A tactile image acquired during the VTI probe elevation (Test 2) with anatomical landmarks and pressure values at specified locations (see A1-A3 and P1-P3 in Figure 2) along anterior and posterior compartments. The VTI software automatically identified all these 6 locations and shows the pressure values and gradient values (nor shown) for these locations.

Figure 3 provides an example of VTI Test 1 results. Figure 4 presents Test 2 results for another subject. The same locations specified in Test 2 for capturing pressure values (parameters 7 – 12 in Table 2) are used for calculation of pressure gradients (parameters 13 – 18 in Table 2).

**Figure 5.**
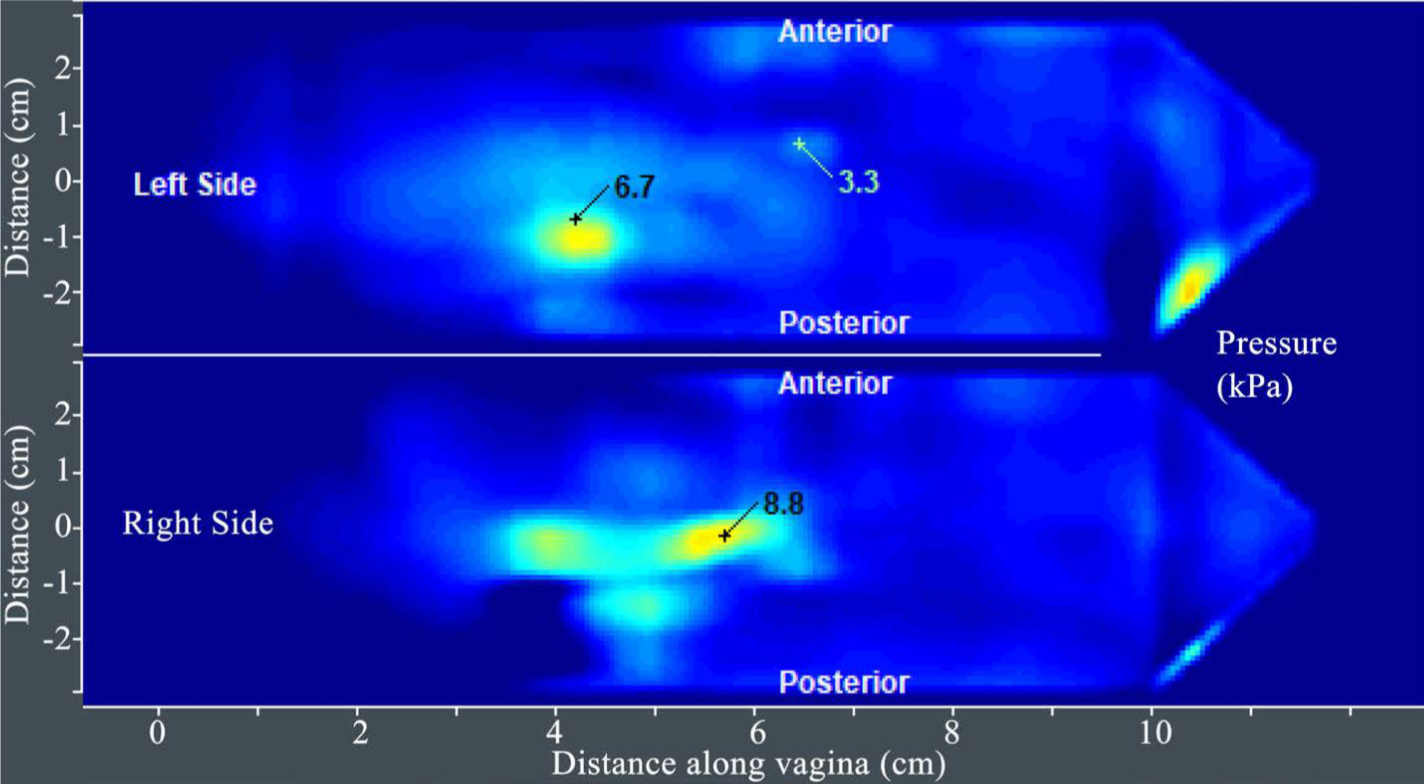
A tactile image acquired during the VTI probe rotation (Test 3) with pressure values at specified locations (see S1 and S2 in Figure 2). The VTI software automatically identified all these 3 locations and shows the pressure values (local maximums) for these locations.

Figure 5 presents Test 3 results for another subject. Test 3 provides 6 pressure values (parameters 19 – 24 in Table 2).

**Figure 6.**
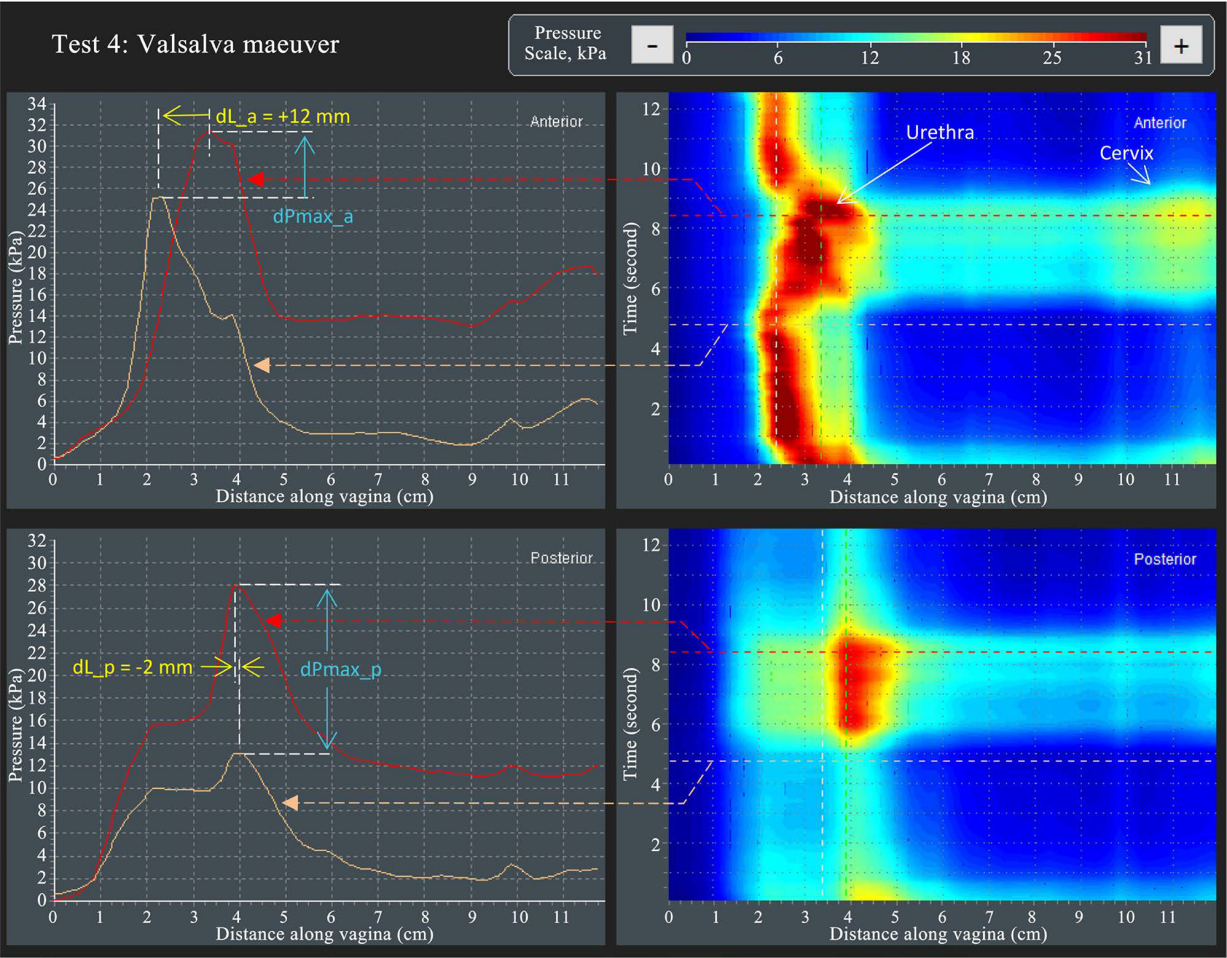
A dynamic pressure patterns acquired during the Valsalva maneuver for anterior and posterior compartments (Test 4).

Figure 6 shows the approach for VTI capturing parameter dL_a (see parameter 27 in Table 2) and dL_p (see parameter 30 in Table 2)—displacements of the maximum pressure peaks in anterior and posterior compartments in Test 4. It also illustrates the approach for VTI capturing parameter dPmax_a (see parameter 26 in Table 2) and dPmax_p (see parameter 29 in Table 2)—changes of maximum pressure peaks at Valsalva maneuver. Please pay attention to the measured dL_a and dL_p which have different sign/direction for this specific subject.

**Figure 7.**
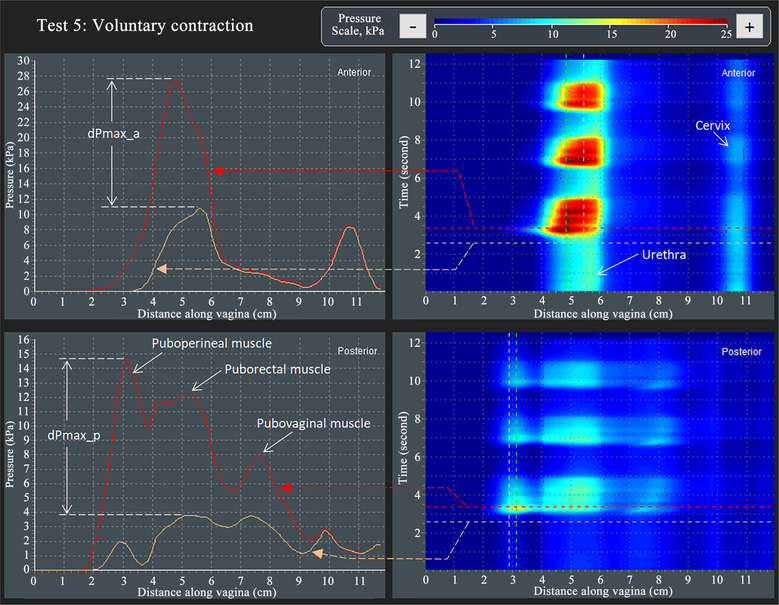
A dynamic pressure patterns acquired during the voluntary muscle contraction for anterior and posterior compartments (Test 5).

Figure 7 illustrates the approach for VTI capturing parameter dPmax_a (see parameter 32 in Table 2) and dPmax_p (see parameter 35 in Table 2)—changes of maximum pressure peaks at voluntary muscles contractions in Test 7. Three contractive peaks are observed in the posterior compartment which are described as originating from puboperineal, puborectal, and pubovaginal muscles. The contractive changes for these 3 posterior peaks have different value and separated along the vagina for this specific subject.

**Figure 8.**
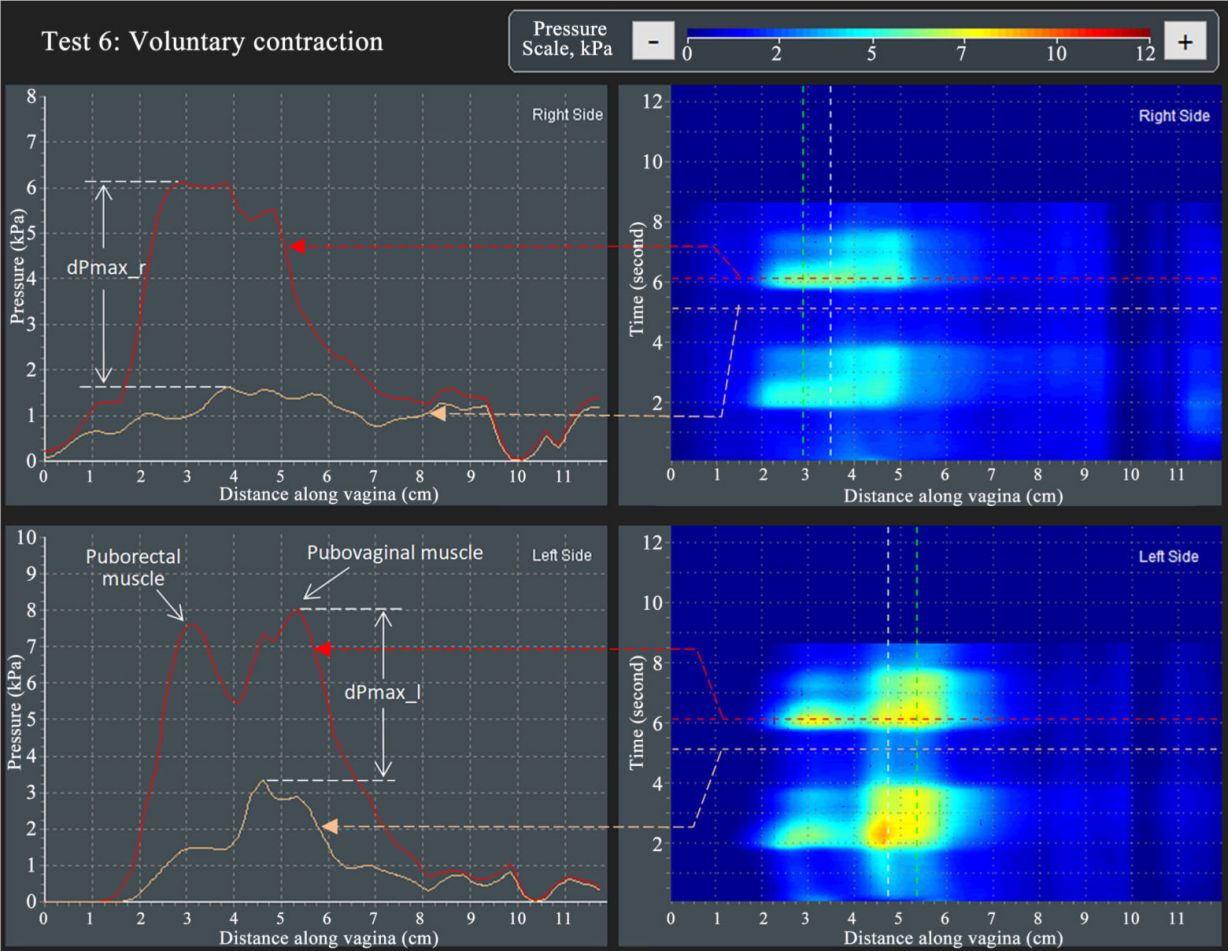
A dynamic pressure patterns acquired during the voluntary muscle contraction for left and right vaginal compartments (Test 6).

Figure 8 illustrates the approach for VTI capturing parameter dPmax_r (see parameter 38 in Table 2) and dPmax_l (see parameter 41 in Table 2)—changes of maximum pressure peaks at voluntary muscles contractions in the right and left vaginal compartments in Test 6. Two contractive peaks are observed per compartment which are identified as puborectal and pubovaginal muscle contractions. The contractive changes for the two sides have differences and are separated along the vagina in the left compartment for this specific subject.

**Figure 9.**
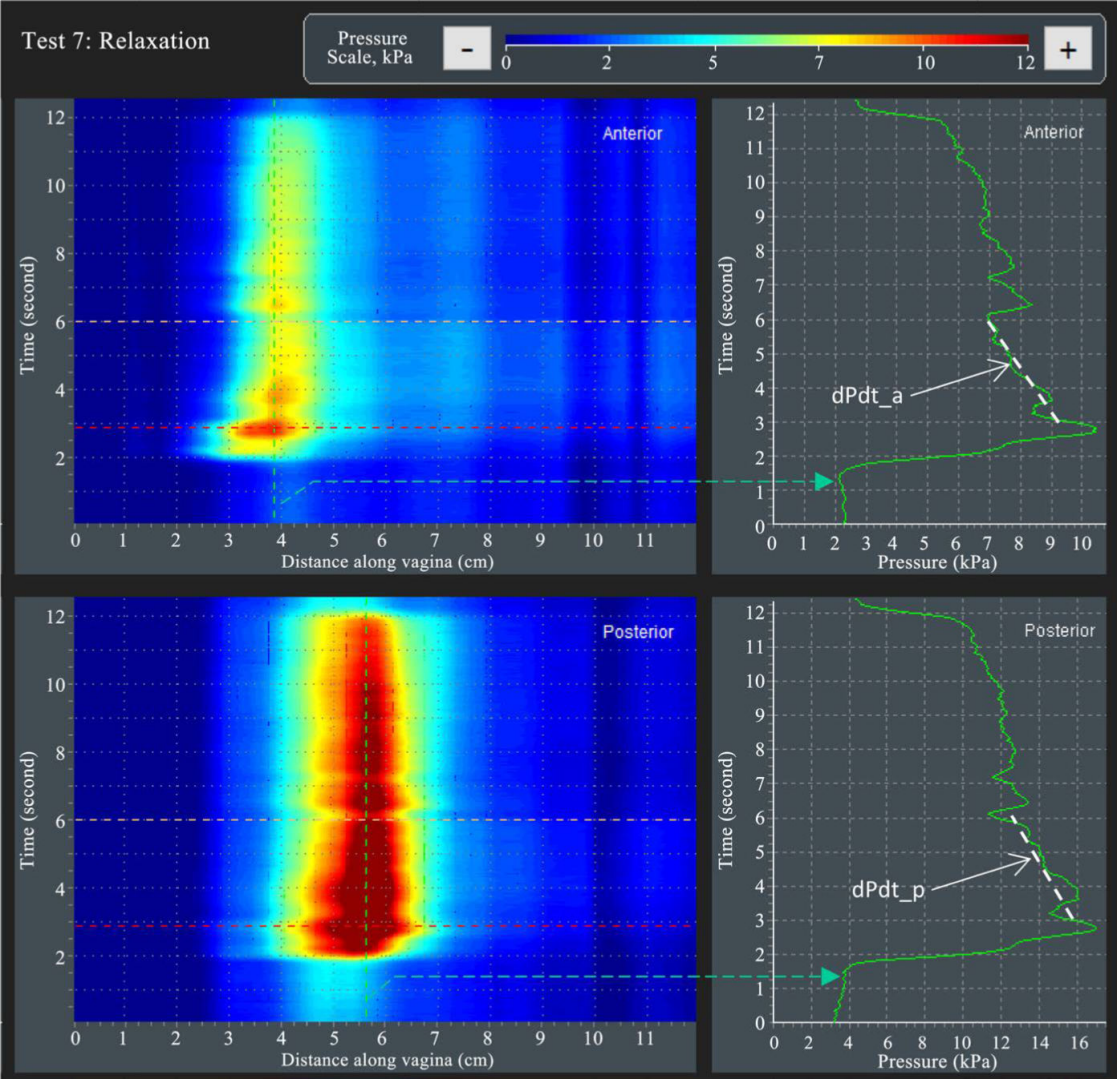
A dynamic pressure patterns acquired during the involuntary muscle relaxation for interior and posterior compartments (Test 7).

Figure 9 illustrates the approach for VTI capturing parameters dPdt_a, dpcdt_a (see parameter 43, 44 in Table 2) and dPdt_p, dpcdt_p (see parameter 45, 46 in Table 2)—absolute and relative (in %) slopes approximated by the white dashed lines for anterior and posterior compartments within three seconds in Test 7. The VTI software captures the relaxation at a location with maximum pressure and calculates the slope in time for this fixed location in the vagina.

**Figure 10.**
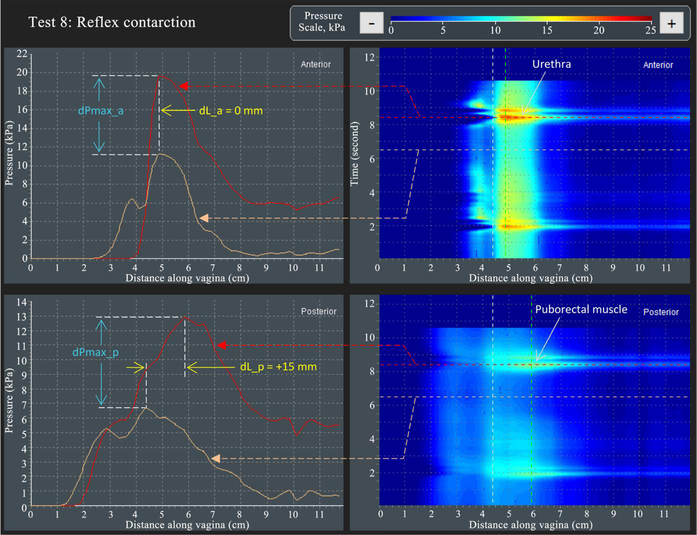
A dynamic pressure patterns acquired during the reflex contraction (cough) for anterior and posterior compartments (Test 8).

Figure 10 shows the approach for VTI capturing parameter dL_a (see parameter 49 in Table 2) and dL_p (see parameter 52 in Table 2)—displacement of the maximum pressure peaks in anterior and posterior compartments during the reflex (involuntary) muscle contraction (cough) in Test 8. It also illustrates the approach for VTI capturing parameter dPmax_a (see parameter 48 in Table 2) and dPmax_p (see parameter 51 in Table 2)—changes of maximum pressure peaks at the reflex contraction. Please note that the measured dL_a = 0 mm and dL_p = +15 mm for this specific subject.

Table 3 displays the calculated statistics (hypothesis testing outcome *H*- and *p*-value) for POP *versus* normal (Norm) conditions, average (Aver) values for 52 biomechanical parameters, standard deviations (SD), and the ranges (Min, Max) for both POP group (54 subjects) and normal group (42 subjects).

Table 4 presents the calculated statistics (hypothesis testing outcome *H*- and *p*-value) for POP *versus* normal (Norm) conditions, average (Aver) values for 52 biomechanical parameters, standard deviations (SD), and the ranges (Min, Max) for both POP group (44 subjects) and normal group (39 subjects) post the age equalization (alignment) of the groups.

Table 5 presents the calculated statistics (hypothesis testing outcome *H*- and *p*-value) for POP *versus* normal (Norm) conditions, average (Aver) values for 52 biomechanical parameters, standard deviations (SD), and the ranges (Min, Max) for both POP group (42 subjects), and normal group (31 subjects) after the parity and age equalization of the groups.

The *t*-tests for the POP group of 54 subjects *versus* a normal group of 42 subjects demonstrate that 33 out of 52 parameters have statistically significant differences between the groups and these parameters have the potential to be used for detection and description of POP conditions. The analyzed groups have the same subject height and weight distributions. At the same time, these primary analyzed groups have differences in age and parity (see Table 3). To explore the possible influence of these differences, both groups were equalized by age. The *t*-tests outcomes and the accompanying data for the POP group of 44 subjects *versus* the normal group of 39 subjects demonstrate that 30 of 52 parameters have statistically significant differences for the groups equalized by age (see Table 4). Furthermore, the primary groups were equalized by parity and age. The *t*-tests outcomes and the accompanying data for the POP group of 42 subjects *versus* the normal group of 31 subjects demonstrates that 29 of 52 parameters have statistically significant differences for the groups equalized by parity and age (see Table 5).

**Figure 11.**
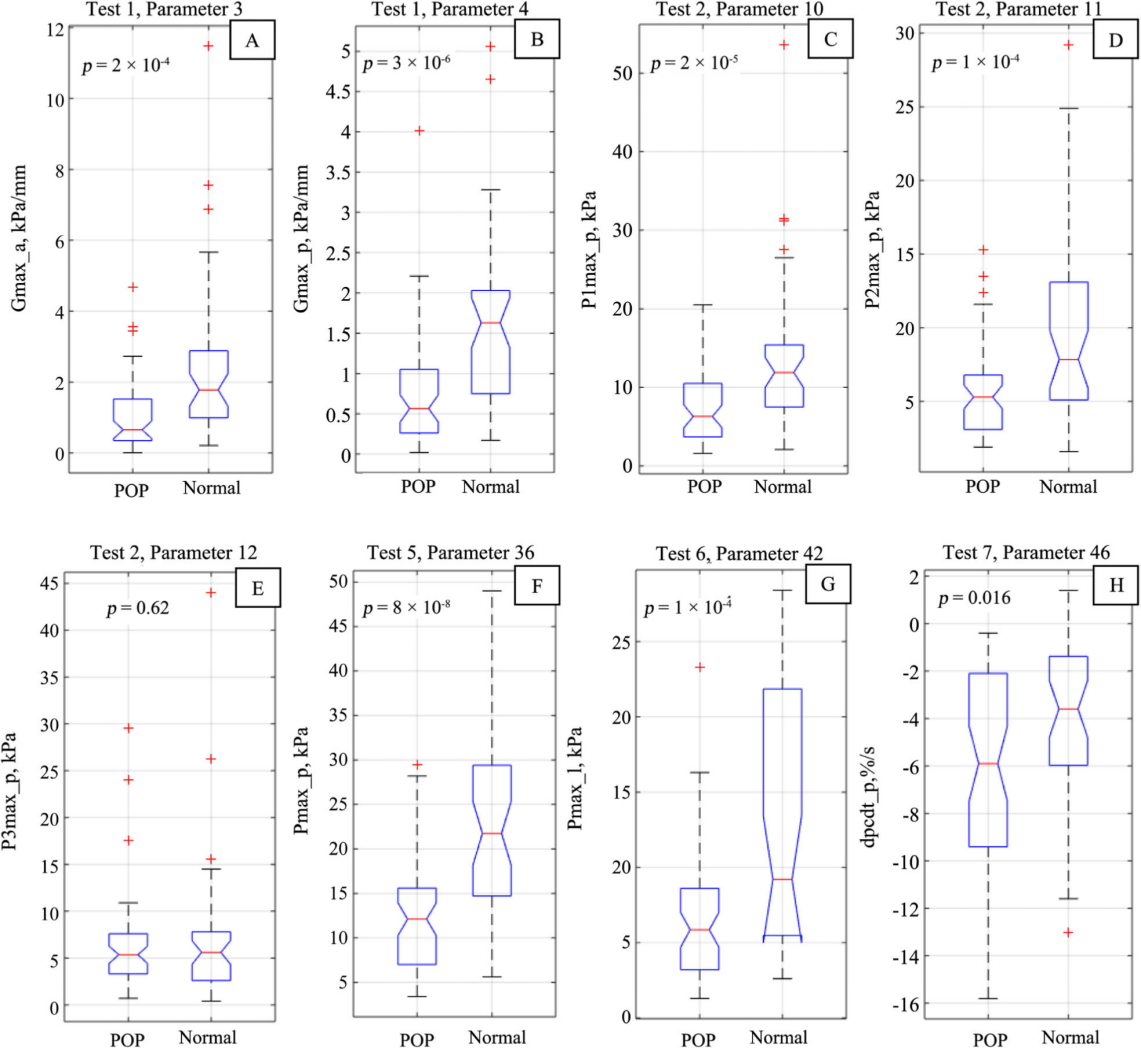
Boxplots A - F for selected biomechanical parameters for POP versus Normal groups from Table 3.

Figure 11 displays the boxplots for selected parameters for POP versus Normal groups presented in Table 3.

## 4. Discussion

The results of this research are in agreement with previously reported data [3]-[10]; however, the current analysis includes the biggest VTI parameter set ever considered. 33 of 52 biomechanical parameters are identified as statistically significant sensitivity to POP *versus* normal pelvic conditions (see Table 3). Their average changes from 39.7% to 145% (82% in average). These changes with POP clearly outperform possible deviations related to VTI intra- and inter-operator variability which were found on an average of ±15.1% (intra-observer error) and ±18.4 (inter-observer error) [7]. These reproducibility errors have intrinsically value and sign by a chance, but we have identified statistically systematical parameter changes with the POP.

Test 1 provides six identified parameters (1, 2, 3, 4, 5, 6) related to tissue elasticity; their average values change from 40.8% to 145% for normal relative to POP conditions. Test 2 provides eight identified parameters (7, 8, 10, 11, 14, 15, 16, 17) related to the pelvic support structure; their average values change from 52.3% to 110.8% for normal relative to POP conditions. Test 3 provides five identified parameters (19, 20, 22, 23, 24) related to tissue elasticity; their average values change from 57.2% to 113.2% for normal relative to POP conditions. Test 4 provides one identified parameters (26) related to pelvic function; its value changes by 67.7 for normal relative to POP conditions. Test 5 provides six identified parameters (31, 32, 33, 34, 35, 36) related to pelvic function; their average values change from 39.7% to 82.1% for normal relative to POP conditions. Test 6 provides four identified parameters (38, 39, 41, 42) related to pelvic function; their average values change from 76.3% to 100.3% for normal relative to POP conditions. Test 7 provides two identified parameters (44, 46) related to pelvic function; their average values change from 50.2% to 103.0% for POP relative to normal conditions. Test 8 provides 1 identified parameter (48) related to pelvic function; its value changes by 69.6% for POP relative to normal conditions. In total, among the 33 identified POP diagnostic parameters, 11 parameters are related to tissue elasticity, 8 parameters are related to pelvic support structures, and 14 parameters are related for to pelvic functions.

The analyzed groups of subjects have differences in age and parity as seen in Table 3. However, after these group equalization by age, 30 of 52 parameters are identified as statistically significant sensitivity to POP *versus* normal pelvic conditions (see Table 4). After these group equalization by parity and age, 29 of 52 parameters are identified as statistically significant sensitivity to POP *versus* normal pelvic conditions (see Table 5). It is important to note that the group with the normal pelvic conditions (no POP, no SUI) was composed of the visitors of urogynecological site; these patients may have some pelvic floor conditions that are not identified in this study. Possibly, the patients from the normal group have had pre-prolapse conditions which haven’t transformed yet into anatomically visible POP. This study reasonably proposes that if the normal group would be composed of 20 – 40 y.o. subjects with no history of consulting urogynecological clinics, more significant differences for the VTI parameters *versus* the POP group may be observed.

The boxplots for selected parameter distributions in Figure 11 display (a) significant tissue elasticity changes with POP (see panels A and B), significant changes with POP in Level III and Level II supports (see panels C and D), but no change in Level I support under POP conditions (see panel E), significant changes in pelvic muscle contractive capabilities with POP (see panels F and G), and significant changes in pelvic muscle relaxation, which related with muscle innervations, with POP development (see panel H).

The next step (which falls beyond the purview of this article) with these biomechanical parameters may include 1) an insight into POP classes (anterior *vs* posterior *vs* uterine), 2) analysis for continence *versus* incontinence conditions, 3) analysis of urogynecological surgical outcomes as a whole as well as per specific surgical procedure, 4) combining the VTI data with urodynamics, ultrasound, and MRI data, 5) to use the VTI and other clinically related data for predicative modeling of outcomes for conservative and surgical procedures (personalized predictive treatment), and 6) maintaining the objective history of biomechanical transformation of the patient pelvic floor.

One of the strengths of this study is that the current VTI offers an opportunity to assess the tissue elasticity, pelvic support structure, and pelvic function (muscle and ligaments) in high definition along the entire length of the anterior, posterior, and lateral walls at rest, with applied deflection pressures and with pelvic muscle contractions. All 52 parameters are calculated automatically in real-time. This allows a large body of measurements to evaluate individual variations in support defects as well as identify specific problematic structures. In addition, the technology provides the opportunity to measure pelvic floor muscle strength at specific locations along the vaginal wall and helps correlate the relative contributions to measured tissue properties. These measurements may provide insight into the functional contribution or relationships between support tissues and the underlying muscle support. Because VTI testing is relatively easy and inexpensive to obtain, post-treatment follow-up is available to evaluate the surgical impact on functional tissue properties and pelvic floor muscles. This may provide valuable outcome measurements for evaluating current and future treatments.

One of the shortcomings of this study is its relatively small sample size. Further studies with larger patient population, investigating a variety of other pelvic floor conditions, and their use in the evaluation of interventions including physical therapy, conservative management options, and surgical correction are needed at this point to further explore the diagnostic values of the biomechanical mapping of the female pelvic floor.

## 5. Conclusion

The biomechanical mapping of the female pelvic floor with the VTI provides a unique set of parameters characterizing POP *versus* normal conditions. These objectively measurable biomechanical transformations of pelvic tissues, support structures and functions under POP may be used in the future research and practical applications.

## Figures and Tables

**Table 1.** VTI Examination inlcudes 8 procedure tests.

Test No.	Procedure	Output
Test 1	Probe insertion	Tactile image for vaginal anterior and posterior compartments along the entire vagina (resistance, force, work, tissue elasticity).
Test 2	Probe elevation	Tactile image for anterior and posterior compartments which related to pelvic floor support structures (pressure value sand pressure gradients for specified/critical locations).
Test 3	Probe rotation	Tactile images for center and right sides along the entire vagina (force and pressure values for specified positions/locations).
Test 4	Valsalva maneuver	Dynamic pressure response from opposite sites (anterior *vs* posterior) along the entire vagina (changes in force and pressure; pressure peak displacements).
Test 5	Voluntary muscle contraction	Dynamic pressure response from opposite sites (anterior *vs* posterior) along the entire vagina (changes in force and pressure; maximum pressure values).
Test 6	Voluntary muscle contraction (sides)	Dynamic pressure response from opposite sides (center *vs* right) along the entire vagina (changes in force and pressure; maximum pressure values).
Test 7	Involuntary relaxation	Dynamic pressure response from opposite sites (anterior *vs* posterior) along the entire vagina (changes in pressure).
Test 8	Reflex muscle contraction (cough)	Dynamic pressure response from opposite sites (anterior *vs* posterior) along the entire vagina (changes in force and pressure; pressure peak displacements).

**Table 2.** VTI biomechanical parameters.

No.	VTI Test	Parameters Abbreviation	Units	Parameter Description	Parameter Interpretation	Parameter Class	Targeting/ Contributing Pelvic Structures
1	1	Fmax	N	Maximum value of force measured during the VTI probe insertion [9]	Maximum resistance of anterior *vs* posterior widening; tissue elasticity at specified location (capability to resist to applied deformation)	Maximum vaginal tissue elasticity at specified location	Tissues behind the anterior and posterior vaginal walls at 3 – 15 mm depth
2	1	Work	mJ	Work completed during the probe insertion (Work = Force × Displacement)[9]	Integral resistance of vaginal tissue (anterior and posterior) along the probe insertion	Average vaginal tissue elasticity	Tissues behind the anterior and posterior vaginal walls at 3 – 15 mm depth
3	1	Gmax_a	kPa/mm	Maximum value of anterior gradient (change of pressure per anterior wall displacement in orthogonal direction to the vaginal channel)	Maximum value of tissue elasticity in anterior compartment behind the vaginal at specified location	Maximum value of anterior tissue elasticity	Tissues/structures in anterior compartment at 10 – 15 mm depth
4	1	Gmax_p	kPa/mm	Maximum value of posterior gradient (change of pressure per posterior wall displacement in orthogonal direction to the vaginal channel)	Maximum value of tissue elasticity in posterior compartment behind the vaginal at specified location	Maximum value of posterior tissue elasticity	Tissues/structures in anterior compartment at 10 – 15 mm depth
5	1	Pmax_a	kPa	Maximum value of pressure per anterior wall along the vagina	Maximum resistance of anterior tissue to vaginal wall deformation	Anterior tissue elasticity	Tissues/structures in anterior compartment
6	1	Pmax_p	kPa	Maximum value of pressure per posterior wall along the vagina	Maximum resistance of posterior tissue to vaginal wall deformation	Posterior tissue elasticity	Tissues/structures in posterior compartment
7	2	P1max_a	kPa	Maximum pressure at thearea of pubic bone (anterior, A1 in Figure 2)	Proximity of pubic bone to vaginal wall and perineal body strength	Anatomic aspects and tissue elasticity	Tissues between vagina and pubic bone; perineal body
8	2	P2max_a	kPa	Maximum pressure at the area of urethra (anterior, A2 in Figure 2)	Elasticity/mobility of urethra	Anatomic aspects and tissue elasticity	Urethra and surrounding tissues
9	2	P3max_a	kPa	Maximum pressure at the cervix area (anterior, A3 in Figure 2)	Mobility of uterus and conditions of uterosacral Uterosacral and cardinal and cardinal ligaments	Pelvic floor support	Uterosacral and cardinal ligaments
10	2	P1max_p	kPa	Maximum pressure at the perineal body (posterioir, see P1 in Figure 2)	Pressure feedback of Level III support	Pelvic floor support	Puboperineal, puborectal muscles
11	2	P2max_p	kPa	Maximum pressure at middle third of vagina (posterioir, see P2 in Figure 2)	Pressure feedback of Level II support	Pelvic floor support	Pubovaginal, puboanal muscles
12	2	P3max_p	kPa	Maximum pressure at upper third of vagina (posterioir, see P3 in Figure 2)	Pressure feedback of Level I support	Pelvic floor support	Iliococcygeal muscle, levator plate
13	2	G1max_a	kPa/mm	Maximum gradient at the area of pubic bone (anterior, see A1 in Figure 2)	Vaginal elasticity at pubic bone area	Anterior tissue elasticity	Tissues between vagina and pubic bone; perineal body
14	2	G2max_a	kPa/mm	Maximum gradient at the area of urethra (anterior, see A2 in Figure 2)	Mobility and elasticity of urethra	Urethral tissue elasticity	Urethra and surrounding tissues
15	2	G3max_a	kPa/mm	Maximum gradient at the cervix area (anterior, see A3 in Figure 2)	Conditions of uterosacral and cardinal ligaments	Pelvic floor support	Uterosacral and cardinal ligaments
16	2	G1max_p	kPa/mm	Maximum gradient at the perineal body (posterioir, see P1 in Figure 2)	Strength of Level III support (tissue deformation up to 25 mm)	Pelvic floor support	Puboperineal, puborectal muscles
17	2	G2max_p	kPa/mm	Maximum gradient at middle third of vagina (posterioir, see P2 in Figure 2)	Strength of Level II support (tissue deformation up to 35 mm)	Pelvic floor support	Pubovaginal, puboanal muscles
18	2	G3max_p	kPa/mm	Maximum gradient at upper third of vagina (posterioir, see P3 in Figure 2)	Strength of Level I support (tissue deformation up to 45 mm)	Pelvic floor support	Iliococcygeal muscle, levator plate
19	3	Pmax	kPa	Maximum pressure at vaginal walls deformation by 7 mm [9]	Hard tissue or tight vagina	Vaginal tissue elasticity	Tissues behind the vaginal walls at 5 – 7 mm depth
20	3	Fap	N	Force applied by anterior and posterior compartments to the probe[9]	Integral strength of anterior and posterior compartments	Vaginal tightening	Tissues behind anterior/ posterior vaginal walls.
21	3	Fs	N	Force applied by entire left and right sides of vagina to the probe[9]	Integral strength of left and right sides of vagina	Vaginal tightening	Vaginal right/left walls and tissues behind them.
22	3	P1_l	kPa	Pressure response from a selected location (irregularity 1) at left side (see S1 in Figure 2)	Hard tissue on left vaginal wall	Irregularity on vaginal wall	Tissue/muscle behind the vaginal walls on left side.
23	3	P2_l	kPa	Pressure response from a selected location (irregularity 2) at left side (see S2 in Figure 2)	Hard tissue on left vaginal wall	Irregularity on vaginal wall	Tissue/muscle behind the vaginal walls on left side.
24	3	P3_r	kPa	Pressure response from a selected location (irregularity 3) at right sidevaginal (see S1 in Figure 2)	Hard tissue on right wall	Irregularity on vaginal wall	Tissue/muscle behind the vaginal walls on right side.
25	4	dF_a	N	Integral force change in anterior compartment at Valsalva maneuver	Pelvic function* at Valsalva maneuver	Pelvic function	Multiple pelvic muscle*
26	4	dPmax_a	kPa	Maximum pressure changt in anterior compartment at Valsalva maneuver.	Pelvic function* at Valsalva maneuver	Pelvic function	Multiple pelvic muscle*
27	4	dL_a	mm	Displacement of the maximum pressure peak instructures* anterior compartment	Mobility of anterior Valsalva maneuver	Pelvic function	Urethra, pubovaginal muscle; ligaments*
28	4	dF_p	N	Integral force change in posterior compartment at Valsalva maneuver	Pelvic function* at Valsalva maneuver	Pelvic function	Multiple pelvic muscle*
29	4	dPmax_p	kPa	Maximum pressure change in posterior compartment at Valsalva maneuver.	Pelvic function* at Valsalva maneuver	Pelvic function	Multiple pelvic muscle*
30	4	dL_p	mm	Displacement of the maximum pressure peak instructures*	Mobility of posterioir Valsalva	Pelvic function	Anorectal, puborectal, pubovaginal muscles;ligaments*
31	5	dF_a	N	Integral force change in anterior compartment at voluntary muscle contraction	Integral contraction strength of pelvic muscles along the vagina	Pelvic function	Puboperineal, puborectal, pubovaginal and ilicoccygeal muscles; uretra
32	5	dPmax_a	kPa	Maximum pressure change in anterior compartment at voluntary muscle contraction	Contraction strength of specified pelvic muscles	Pelvic function	Puboperineal, puborectal and pubovaginal muscles
33	5	Pmax_a	kPa	Maximum pressure value in anterior compartment at voluntary muscle contraction.	Static and dynamic peak support of the pelvic floor	Pelvic function	Puboperineal and puborectal muscles*
34	5	dF_p	N	Integral force change in posterior compartment at voluntary muscle contraction	Integral contraction strength of pelvic muscles along the vagina	Pelvic function	Puboperineal, puborectal, pubovaginal and ilicoccygeal muscles
35	5	dPmax_p	kPa	Maximum pressure change in posterior compartment at voluntary muscle contraction	Contraction strength of pelvic muscles at specified location	Pelvic function	Puboperineal, puborectal and pubovaginal muscles
36	5	Pmax_p	kPa	Maximum pressure value in posterior compartment at voluntary muscle contraction.	Static and dynamic peak support of the pelvic floor	Pelvic function	Puboperineal and puborectal muscles*
37	6	dF_r	N	Integral force change in right side at voluntary muscle contraction	Integral contraction strength of pelvic muscles along the vagina	Pelvic function	Puboperineal, puborectal, and pubovaginal muscles
38	6	dPmax_r	kPa	Maximum pressure change in right side at voluntary muscle contraction	Contraction strength of specific pelvic muscle	Pelvic function	Puboperineal or puborectal or pubovaginal muscles
39	6	Pmax_r	kPa	Maximum pressure value in right side at voluntary muscle contraction	Specified pelvic muscle contractive capability and integrity	Pelvic function	Puboperineal or puborectal muscles
40	6	dF_l	N	Integral force change in left side at voluntary muscle contraction	Integral contraction strength of pelvic muscles along the vagina	Pelvic function	Puboperineal, puborectal, and pubovaginal muscles
41	6	dPmax_l	kPa	Maximum pressure change in left side at voluntary muscle contraction	Contraction strength of specific pelvic muscle	Pelvic function	Puboperineal or puborectal or pubovaginal muscles
42	6	Pmax_l	kPa	Maximum pressure value in left side at voluntary muscle contraction	Specified pelvic muscle contractive capability and integrity	Pelvic function	Puboperineal or puborectal muscles
43	7	dPdt_a	kPa/s	Anterior absolute pressure change per second for maximum pressure at involuntary relaxation	Innervation status of specified pelvic muscles	Innervations status	Levator ani muscles
44	7	dpcdt_a	%/s	Anterior relative pressure change per second for maximum pressure at involuntary relaxation	Innervation status of specified pelvic muscles	Innervations status	Levator ani muscles
45	7	dPdt_p	kPa/s	Posterior absolute pressure change per second for maximum pressure at involuntary relaxation	Innervation status of specified pelvic muscles	Innervations status	Levator ani muscles
46	7	dpcdt_p	%/s	Posterior relative pressure change per second for maximum pressure at involuntary relaxation	Innervation status of specified pelvic muscles	Innervations status	Levator ani muscles
47	8	dF_a	N	Integral force change in anterior compartment at reflex pelvic muscle contraction (cough)	Integral pelvic function* at reflex muscle contraction	Pelvic function	Multiple pelvic muscle*
48	8	dPmax_a	kPa	Maximum pressure change in anterior compartment at reflex pelvic muscle contraction (cough).	Contraction strength of specified pelvic muscles	Pelvic function	Multiple pelvic muscle*
49	8	dL_a	mm	Displacement of the maximum pressure peak instructures* anterior compartment	Mobility of anterior at reflex muscle contraction	Pelvic function	Urethra, pubovaginal muscle; ligaments*
50	8	dF_p	N	Integral force change in posterior compartment at reflex pelvic muscle contraction (cough)	Integral pelvic function* at reflex muscle contraction	Pelvic function	Multiple pelvic muscle*
51	8	dPmax_p	kPa	Maximum pressure change in posterior compartment at reflex pelvic muscle contraction (cough).	Contraction strength of specified pelvic muscles	Pelvic function	Multiple pelvic muscle*
52	8	dL_p	mm	Displacement of the maximum pressure peak instructures* posterior compartment	Mobility of anterior at reflex muscle contraction	Pelvic function	Anorectal, puborectal and pubovaginal muscles; ligaments*

* requires further interpretation.

**Table 3.** Biomechanical Parameters: Prolapse (group of 54 subjects) versus Normal conditions (group of 42 subjects).

		*H*	*p*	Units	Aver POP	Aver Norm	SD POP	SD Norm	Min POP	Min Norm	Max POP	Max Norm
		0	0.828	cm	162.1	161.7	7.0	11.8	150	124	178	180
Height→		0	0.311	Ib	157.4	151.2	31.8	26.4	105	110	243	200
Weight→												
Age→		1	0.005	y.o.	59.0	51.2	10.6	16.0	37	26	82	90
Parity (P)→		1	9 × 10^−5^ 8.76E-05	-	2.5	1.4	1.1	1.0	0	0	6	3
Parameters number↓	Test↓											
1	1	1	5 × 10^−5^	N	0.73	1.24	0.44	0.74	0.22	0.23	2.74	4.05
2	1	1	0.001	mJ	30.06	42.34	13.91	22.46	9.50	4.50	68.10	96.30
3	1	1	2 × 10^−4^	kPa/mm	1.06	2.38	0.98	2.21	0.01	0.21	4.69	11.48
4	1	1	3 × 10^−5^	kPa/mm	0.77	1.57	0.70	1.08	0.02	0.17	4.02	5.06
5	1	1	1 × 10^−7^	kPa	16.09	39.43	11.30	26.78	3.10	6.00	52.10	145.50
6	1	1	8 × 10^−6^	kPa	11.70	22.64	8.06	14.33	3.20	5.10	46.70	60.90
7	2	1	0.001	kPa	18.54	28.24	13.76	15.13	1.60	4.50	57.10	70.50
8	2	1	3 × 10^−6^	kPa	6.00	11.85	3.43	7.72	1.90	0.10	20.10	31.80
9	2	0	0.082	kPa	5.88	8.51	6.66	8.03	0.80	0.00	50.30	40.70
10	2	1	2 × 10^−5^	kPa	7.11	13.80	4.44	9.65	1.60	2.10	20.50	53.60
11	2	1	1 × 10^−4^	kPa	5.52	9.54	3.10	6.41	1.90	1.60	15.30	29.20
12	2	0	0.620	kPa	6.30	6.94	5.01	7.62	0.70	0.40	29.60	44.00
13	2	0	0.254	kPa/mm	1.53	1.89	1.35	1.66	0.05	0.00	5.60	6.15
14	2	1	0.002	kPa/mm	0.38	0.79	0.35	0.84	0.03	0.00	1.70	3.95
15	2	1	0.010	kPa/mm	0.28	0.57	0.42	0.67	0.01	0.00	2.54	3.30
16	2	1	0.006	kPa/mm	0.35	0.73	0.38	0.90	0.01	0.06	2.11	4.91
17	2	1	0.004	kPa/mm	0.25	0.41	0.23	0.30	0.01	0.05	1.37	1.16
18	2	0	0.204	kPa/mm	0.31	0.44	0.35	0.60	0.05	0.00	1.80	3.48
19	3	1	2 × 10^−6^	kPa	16.67	32.16	14.19	15.51	4.16	5.04	62.40	69.40
20	3	1	1 × 10^−5^	N	2.54	4.03	1.27	1.91	0.78	1.26	6.55	9.15
21	3	0	0.716	N	1.24	1.19	0.64	0.83	0.17	0.10	3.12	3.49
22	3	1	3 × 10^−5^	kPa	4.71	9.21	3.66	6.36	1.00	2.30	22.10	30.70
23	3	1	5 × 10^−4^	kPa	3.14	4.93	1.65	3.14	0.90	0.80	10.10	12.90
24	3	1	5 × 10^−7^	kPa	4.62	9.86	2.77	6.43	1.10	2.40	12.40	25.50
25	4	0	0.157	N	1.52	1.24	0.96	0.80	0.17	0.31	4.64	3.78
26	4	1	0.039	kPa	6.34	10.63	8.30	10.81	−14.70	−4.30	40.90	40.20
27	4	0	0.071	mm	4.92	1.83	9.03	5.02	−19.00	−12.30	27.80	13.50
28	4	0	0.125	N	1.53	1.22	0.93	0.89	0.16	0.05	4.43	4.07
29	4	0	0.364	kPa	5.81	6.80	4.14	6.06	0.30	0.20	18.20	21.60
30	4	0	0.551	mm	3.14	2.39	5.92	5.52	−7.00	−10.00	22.80	18.80
31	5	1	0.007	N	1.09	1.57	0.73	0.99	0.13	0.30	3.12	5.89
32	5	1	0.043	kPa	15.94	22.27	14.57	15.44	0.30	1.80	56.50	80.40
33	5	1	7 × 10^−5^	kPa	24.95	40.86	18.12	19.26	3.80	4.40	76.00	99.40
34	5	1	0.001	N	1.15	1.84	0.77	1.27	0.20	0.31	3.53	5.87
35	5	1	1 × 10^−5^	kPa	7.72	13.86	5.16	9.62	0.50	2.00	20.60	44.40
36	5	1	8 × 10^−8^	kPa	12.49	22.75	6.33	10.75	3.40	5.60	29.50	49.00
37	6	0	0.077	N	0.63	0.85	0.55	0.64	0.03	0.09	2.62	2.77
38	6	1	7 × 10^−4^	kPa	4.01	7.68	3.93	6.11	0.10	0.20	18.20	23.60
39	6	1	7 × 10^−6^	kPa	6.65	13.32	4.94	8.35	1.30	2.20	22.70	29.50
40	6	0	0.162	N	0.66	0.85	0.59	0.71	0.02	0.09	3.07	3.18
41	6	1	0.003	kPa	3.93	6.92	3.70	5.54	0.04	0.50	16.90	20.60
42	6	1	1 × 10^−4^	kPa	6.88	12.37	4.54	8.28	1.30	2.60	23.30	28.40
43	7	0	0.563	kPa/s	−1.53	−1.29	2.09	1.63	−9.90	−6.44	0.07	0.72
44	7	1	0.001	%/s	−6.36	−3.10	5.05	3.56	−21.90	−11.70	0.30	4.30
45	7	0	0.363	kPa/s	−0.80	−1.01	0.88	1.35	−4.70	−6.10	−0.02	0.37
46	7	1	0.016	%/s	−6.19	−4.11	4.07	3.84	−15.80	−13.00	−0.40	1.40
47	8	0	0.535	N	2.10	2.26	0.91	1.42	0.57	0.13	4.15	5.53
48	8	1	0.025	kPa	8.22	13.93	8.30	14.94	−23.80	−17.30	31.40	61.50
49	8	0	0.945	mm	6.64	6.52	9.08	4.71	−5.00	−3.50	27.50	17.30
50	8	0	0.901	N	2.28	2.25	1.03	1.50	0.66	0.43	4.96	5.19
51	8	0	0.097	kPa	9.06	11.41	4.79	8.22	2.20	1.00	21.80	27.30
52	8	0	0.342	mm	5.09	3.65	6.96	6.33	−10.00	−5.00	22.30	20.00

**Table 4.** Biomechanical parameters: prolapse (group of 44 subjects) versus Normal conditions (group of 39 subjects). Thesegroups are equalized by age.

		*H*	*p*	Units	Aver POP	Aver Norm	SD POP	SD Norm	MIn POP	Min Norm	Max POP	Max Norm
		0	0.862	cm	162.7	161.5	6.6	11.57	150	125	176	177
Height→		0	0.318	Ib	159.4	153.3	28.4	27.7	105	110	233	200
Weight→												
Age→		0	0.342	y.o	54.1	53.9	7.8	15.3	37	31	65	90
Parity (P)→		1	0.001	-	2.4	1.6	1.1	1.0	1	0	6	3
Parameters number↓	Test↓											
1	1	1	2 × 10^−5^	N	0.71	1.19	0.36	0.74	0.24	0.23	1.67	4.05
2	1	1	0.010	mJ	30.00	40.37	13.64	21.77	10.20	4.50	68.10	96.30
3	1	1	0.002	kPa/mm	1.11	2.27	0.99	2.23	0.01	0.21	4.69	11.48
4	1	1	2 × 10^−4^	kPa/mm	0.77	1.55	0.71	1.12	0.05	0.17	4.02	5.06
5	1	1	6 × 10^−6^	kPa	16.60	38.72	11.65	27.56	3.10	6.00	52.10	145.50
6	1	1	9 × 10^−5^	kPa	11.43	22.07	8.18	14.72	3.20	5.10	46.70	60.90
7	2	1	0.006	kPa	18.82	27.92	14.14	15.45	1.60	4.50	57.10	70.50
8	2	1	1 × 10^−4^	kPa	6.09	10.92	3.56	6.93	1.90	0.10	20.10	28.10
9	2	0	0.19456	kPa	6.06	8.29	7.28	8.23	1.70	0.00	50.30	40.70
10	2	1	0.001	kPa	7.55	12.64	4.52	8.95	2.10	2.10	20.50	53.60
11	2	1	2 × 10^−4^	kPa	5.28	9.07	2.71	5.76	1.90	1.60	13.50	29.20
12	2	0	0.466	kPa	5.54	6.24	3.74	4.96	0.70	0.70	24.00	26.30
13	2	0	0.177	kPa/mm	1.43	1.85	1.16	1.68	0.05	0.00	5.10	6.15
14	2	1	0.010	kPa/mm	0.37	0.73	0.34	0.83	0.03	0.00	1.70	3.95
15	2	1	0.045	kPa/mm	0.29	0.55	0.46	0.68	0.05	0.00	2.54	3.30
16	2	0	0.062	kPa/mm	0.37	0.65	0.41	0.89	0.01	0.06	2.11	4.91
17	2	1	0.001	kPa/mm	0.23	0.39	0.15	0.30	0.01	0.05	0.68	1.16
18	2	0	0.322	kPa/mm	0.29	0.37	0.36	0.38	0.05	0.00	1.80	1.89
19	3	1	1 × 10^−4^	kPa	17.40	30.98	15.09	15.24	4.30	5.04	62.40	69.40
20	3	1	3 × 10^−4^	N	2.54	3.82	1.28	1.79	0.78	1.26	6.55	9.15
21	3	0	0.478	N	1.25	1.13	0.68	0.82	0.17	0.10	3.12	3.49
22	3	1	0.001	kPa	4.93	8.34	3.96	5.33	1.00	2.30	22.10	24.80
23	3	1	0.011	kPa	3.23	4.56	1.78	2.79	0.90	0.80	10.10	11.80
24	3	1	4 × 10^−5^	kPa	4.72	9.24	2.82	6.25	1.10	2.40	12.40	25.50
25	4	1	0.047	N	1.58	1.16	1.02	0.68	0.17	0.31	4.64	3.11
26	4	0	0.152	kPa	6.56	9.74	9.07	9.97	−14.70	−4.30	40.90	40.20
27	4	0	0.145	mm	4.71	2.08	9.17	4.90	−19.00	−12.30	27.80	13.50
28	4	1	0.030	N	1.59	1.13	1.00	0.76	0.16	0.05	4.43	3.05
29	4	0	0.993	kPa	6.31	6.30	4.42	5.63	0.30	0.20	18.20	21.00
30	4	0	0.689	mm	2.76	2.23	5.77	5.52	−7.00	−10.00	22.80	18.80
31	5	0	0.110	N	1.16	1.42	0.76	0.71	0.13	0.30	3.12	3.14
32	5	0	0.469	kPa	17.85	20.05	15.27	11.91	0.30	1.80	56.50	45.70
33	5	1	0.004	kPa	26.96	38.58	18.72	16.82	3.80	4.40	76.00	77.80
34	5	1	0.029	N	1.23	1.70	0.81	1.12	0.20	0.31	3.53	4.83
35	5	1	0.006	kPa	8.48	12.78	5.26	8.43	0.60	2.00	20.60	34.30
36	5	1	2 × 10^−5^	kPa	13.34	21.53	6.32	9.89	3.90	5.60	29.50	43.40
37	6	0	0.393	N	0.67	0.78	0.58	0.55	0.03	0.09	2.62	2.07
38	6	1	0.017	kPa	4.52	7.22	4.15	5.64	0.10	0.20	18.20	21.50
39	6	1	5 × 10^−4^	kPa	7.21	12.77	5.24	8.19	1.30	2.20	22.70	29.50
40	6	0	0.587	N	0.70	0.77	0.62	0.60	0.02	0.09	3.07	2.69
41	6	1	0.038	kPa	4.35	6.53	3.94	5.18	0.10	0.50	16.90	18.70
42	6	1	0.003	kPa	7.32	11.75	4.79	8.05	1.30	2.60	23.30	28.40
43	7	0	0.325	kPa/s	−1.73	−1.27	2.24	1.68	−9.90	−6.44	0.07	0.72
44	7	1	0.001	%/s	−6.53	−3.06	5.00	3.65	−21.90	−11.70	0.30	4.30
45	7	0	0.778	kPa/s	−0.91	−0.98	0.92	1.37	−4.70	−6.10	−0.03	0.37
46	7	1	0.005	%/s	−6.64	−4.04	4.04	3.89	−15.80	−13.00	−0.40	1.40
47	8	0	0.561	N	2.22	2.07	0.88	1.26	0.58	0.13	4.15	5.10
48	8	0	0.131	kPa	8.69	12.89	8.81	14.85	−23.80	−17.30	31.40	61.50
49	8	0	0.518	mm	7.64	6.44	9.23	4.80	−5.00	−3.50	27.50	17.30
50	8	0	0.180	N	2.45	2.07	1.02	1.38	0.66	0.43	4.96	5.19
51	8	0	0.674	kPa	9.96	10.57	4.77	7.74	2.20	1.00	21.80	27.30
52	8	0	0.289	mm	5.15	3.49	7.04	5.81	−10.00	−4.80	22.30	20.00

**Table 5.** Biomechanical parameters: Prolapse (group of 42 subjects) versus Normal conditions (group of 31 subjects). Thesegroups are equalized by parity and age.

		*H*	*p*	Units	Aver POP	Aver Norm	SD POP	SD Norm	MIn POP	Min Norm	Max POP	Max Norm
		0	0.988	cm	161.9	161.9	7.0	11.3	150	125	176	177
Height→		0	0.191	Ib	162.2	152.9	32.9	27.2	105	110	243	200
Weight→												
Age→		0	0.123	y.o	57.7	53.1	9.0	16.1	37	26	75	90
Parity (P)→												
		0	0.968	n/a	1.9	1.9	0.7	0.7	0	1	3	3
Parameters number↓	Test↓											
1	1	1	5 × 10^−4^	N	0.72	1.10	0.37	0.53	0.22	0.26	1.67	2.18
2	1	0	0.056	mJ	31.80	39.29	13.94	19.08	10.20	4.50	68.10	88.60
3	1	1	0.004	kPa/mm	1.09	2.01	1.01	1.66	0.01	0.21	4.69	7.56
4	1	1	0.002	kPa/mm	0.76	1.52	0.74	1.21	0.02	0.17	4.02	5.06
5	1	1	2 × 10^−6^	kPa	16.40	37.09	11.24	22.61	3.10	6.00	52.10	84.50
6	1	1	0.001	kPa	11.82	21.24	8.41	15.14	3.20	5.10	46.70	60.90
7	2	1	0.006	kPa	18.75	29.20	14.33	17.35	1.60	4.50	57.10	70.50
8	2	1	5 × 10^−5^	kPa	6.32	12.42	3.55	8.20	2.20	1.60	20.10	31.80
9	2	0	0.155	kPa	6.27	9.00	7.33	8.92	1.70	0.00	50.30	40.70
10	2	1	0.003	kPa	7.62	12.91	4.68	9.82	1.60	2.10	20.50	53.60
11	2	1	4 × 10^−4^	kPa	5.49	9.67	2.97	6.35	1.90	2.50	13.50	29.20
12	2	0	0.608	kPa	5.81	6.34	3.70	5.11	1.50	0.70	24.00	26.30
13	2	1	0.023	kPa/mm	1.41	2.23	1.24	1.79	0.05	0.00	5.60	6.15
14	2	1	4 × 10^−4^	kPa/mm	0.36	0.92	0.29	0.92	0.03	0.00	1.10	3.95
15	2	1	0.009	kPa/mm	0.27	0.63	0.38	0.76	0.05	0.00	2.54	3.30
16	2	0	0.057	kPa/mm	0.36	0.68	0.41	0.96	0.01	0.06	2.11	4.91
17	2	1	0.005	kPa/mm	0.23	0.38	0.18	0.26	0.01	0.05	0.73	1.16
18	2	0	0.339	kPa/mm	0.29	0.37	0.35	0.36	0.05	0.00	1.80	1.89
19	3	1	2 × 10^−4^	kPa	17.23	31.10	14.57	15.65	4.16	5.04	62.40	69.40
20	3	1	0.002	N	2.69	3.86	1.29	1.89	0.98	1.26	6.55	9.15
21	3	0	0.106	N	1.33	1.05	0.66	0.80	0.53	0.10	3.12	3.49
22	3	1	0.009	kPa	5.06	8.35	4.02	6.36	1.00	2.30	22.10	30.70
23	3	0	0.091	kPa	3.34	4.17	1.78	2.32	1.10	0.80	10.10	11.30
24	3	1	6 × 10^−4^	kPa	4.82	8.67	2.80	6.19	1.30	2.40	12.40	25.50
25	4	0	0.096	N	1.50	1.14	0.95	0.66	0.17	0.33	4.64	3.11
26	4	0	0.087	kPa	6.33	10.48	9.18	10.25	−14.70	−4.30	40.90	40.20
27	4	1	0.042	mm	5.02	1.31	8.26	4.99	−10.00	−12.30	27.80	13.50
28	4	0	0.112	N	1.51	1.16	0.93	0.78	0.16	0.05	4.43	3.05
29	4	0	0.495	kPa	5.91	6.77	4.43	5.86	0.30	0.20	18.20	21.00
30	4	0	0.433	mm	3.21	2.06	5.86	5.79	−3.00	−10.00	22.80	18.80
31	5	1	0.042	N	1.10	1.46	0.72	0.73	0.13	0.30	3.12	3.14
32	5	0	0.378	kPa	16.69	19.46	14.17	11.79	0.30	1.80	54.50	45.70
33	5	1	0.008	kPa	26.08	37.58	18.15	17.14	3.80	4.40	76.00	77.80
34	5	1	0.011	N	1.17	1.72	0.74	1.06	0.22	0.31	3.41	3.90
35	5	1	0.003	kPa	8.13	13.04	5.13	8.36	0.60	2.00	20.60	34.30
36	5	1	3 × 10^−5^	kPa	13.16	21.76	6.33	10.18	3.90	5.60	29.50	43.40
37	6	0	0.511	N	0.65	0.74	0.57	0.51	0.06	0.09	2.62	1.94
38	6	1	0.023	kPa	4.18	6.80	3.97	5.55	0.40	0.20	18.20	21.50
39	6	1	0.002	kPa	7.05	12.11	5.19	8.25	1.40	2.20	22.70	29.50
40	6	0	0.862	N	0.67	0.69	0.60	0.45	0.09	0.09	3.07	1.56
41	6	1	0.047	kPa	3.94	5.97	3.71	4.76	0.20	0.50	16.90	18.40
42	6	1	0.007	kPa	6.91	11.09	4.74	7.82	2.00	2.60	23.30	28.40
43	7	0	0.423	kPa/s	−1.68	−1.29	2.26	1.68	−9.90	−6.44	0.07	0.51
44	7	1	0.001	%/s	−6.69	−3.10	4.81	3.70	−21.90	−11.70	0.30	4.30
45	7	0	0.457	kPa/s	−0.82	−1.03	0.88	1.47	−4.70	−6.10	−0.03	0.37
46	7	1	0.014	%/s	−6.35	−3.96	3.91	3.94	−15.80	−13.00	−0.40	1.40
47	8	0	0.985	N	2.14	2.13	0.89	1.51	0.58	0.13	4.15	5.53
48	8	0	0.159	kPa	7.49	11.74	8.16	16.14	−23.80	−17.30	22.40	61.50
49	8	0	0.561	mm	7.65	6.39	9.80	5.16	−5.00	−3.50	27.50	17.30
50	8	0	0.465	N	2.36	2.12	1.04	1.56	0.66	0.43	4.96	5.19
51	8	0	0.430	kPa	9.36	10.70	4.98	8.78	2.20	1.00	21.80	27.30
52	8	0	0.057	mm	5.60	2.39	7.40	4.32	−10.00	−5.00	22.30	17.50
